# A key role of the subdiaphragmatic vagus nerve in the depression-like phenotype and abnormal composition of gut microbiota in mice after lipopolysaccharide administration

**DOI:** 10.1038/s41398-020-00878-3

**Published:** 2020-06-09

**Authors:** Jiancheng Zhang, Li Ma, Lijia Chang, Yaoyu Pu, Youge Qu, Kenji Hashimoto

**Affiliations:** 1 Division of Clinical Neuroscience, Chiba University Center for Forensic Mental Health, Chiba, 260-8670 Japan; 2 Department of Critical Care Medicine, Union Hospital, Tongji Medical College, Huazhong University of Science and Technology, Wuhan, 430022 PR China

**Keywords:** Depression, Molecular neuroscience

## Abstract

The vagus nerve plays a role in the cross talk between the brain and gut microbiota, which could be involved in depression. The subdiaphragmatic vagus nerve serves as a major modulatory pathway between the brain and gut microbiota. Here, we investigated the effects of subdiaphragmatic vagotomy (SDV) on the depression-like phenotype and the abnormal composition of gut microbiota in mice after lipopolysaccharide (LPS) administration. LPS caused a depression-like phenotype, inflammation, increase in spleen weight, and downregulation of synaptic proteins in the medial prefrontal cortex (mPFC) in the sham-operated mice. In contrast, LPS did not produce a depression-like phenotype and downregulated synaptic proteins in the mPFC after SDV. The spleen weight and plasma levels of pro-inflammatory cytokines in the SDV + LPS group were lower than those of the sham + LPS group. Interestingly, there were positive correlations between the plasma levels of pro-inflammatory cytokines and spleen weight, suggesting a relationship between inflammatory events and spleen weight. Furthermore, LPS led to significant alterations in gut microbiota diversity in sham-operated mice, but not SDV-operated mice. In an unweighted UniFrac PCoA, the dots representing the sham + LPS group were located far away from the dots representing the other three groups. Our results suggest that LPS produces a depression-like phenotype, increases spleen weight, triggers inflammation, downregulates synaptic proteins in the mPFC, and leads to abnormal composition of gut microbiota via the subdiaphragmatic vagus nerve. It is likely that the vagus nerve plays a crucial role in the brain–gut–microbiota axis.

## Introduction

Depression, a common mental disorder, affects 264 million people worldwide and places great pressure on the global burden of disease^1^. Although inflammation plays a crucial role in the pathogenesis of depression^2–8^, the precise mechanisms underlying inflammation-related depression are not fully understood. Meta-analyses showed that patients with depression exhibit higher expression levels of pro-inflammatory cytokines, including interleukin-6 (IL-6) and the tumor necrosis factor-α (TNF-α), compared with healthy control subjects^9–16^.

Interestingly, we reported previously that alterations of peripheral IL-6, but not cerebral IL-6, might lead to resilience rather than susceptibility to inescapable electric stress in a rat model of learned helplessness^17^; moreover, we showed that blockage of the IL-6 receptor in the periphery produced rapid-acting sustained antidepressant effects in a murine model of chronic social defeat stress (CSDS)^18^. Thus, it is likely that inflammation in the periphery plays an important role in depression-like phenotypes in rodents.

The peripheral administration of a bacterial endotoxin (lipopolysaccharide (LPS)) induces depression-like behaviors after triggering inflammation in rodents^2,19^. Therefore, the LPS-induced depression model has been widely used as an inflammatory model of depression in rodents^20–25^. The spleen, which is a large immune organ, plays an essential role in systemic immune function. Recently, we demonstrated a notable increase in splenic size and weight in CSDS-susceptible mice compared with non-CSDS control mice and CSDS-resilient mice^26^, suggesting a role for the brain–spleen axis in stress-induced depression.

Accumulating evidence suggests that the brain–gut–microbiota axis plays an important role in the pathogenesis of depression, as the composition of gut microbiota in patients with depression is altered compared with healthy control subjects^27–29^. Preclinical studies showed that abnormal composition of gut microbiota might contribute to the depression-like behaviors detected in rodents^30–39^. Interestingly, it is suggested that the communication between the brain and the endogenous and exogenous microorganisms in the gut is modulated by the vagus nerve system^40–46^. The ingestion of beneficial bacteria alleviated stress-induced anxiety and depression-like behaviors via the subdiaphragmatic vagus nerve; moreover, these antidepressant-like effects were abolished after subdiaphragmatic vagotomy (SDV)^46^. SDV blocked the depression-like phenotype after intraperitoneal injection of recombinant IL-1β or LPS^47^, suggesting a role for the subdiaphragmatic vagus nerve in LPS-induced depression-like phenotypes in rodents. However, no study has demonstrated the role of the subdiaphragmatic vagus nerve in the effects of the brain–gut–microbiota axis and brain–spleen axis on LPS-induced depression-like phenotype.

Given the key role of inflammation in depression, the present study was undertaken to investigate whether the subdiaphragmatic vagus nerve plays a role in the depression-like phenotype and gut microbiota composition observed in mice after LPS administration. Furthermore, we measured the plasma levels of pro-inflammatory cytokines (i.e., IL-6 and TNF-α) and synaptic proteins [i.e., α-amino-3-hydroxy-5-methyl-4-isoxazolepropionic acid receptor A1 (GluA1) and the postsynaptic density-95 (PSD-95) protein] in the medial prefrontal cortex (mPFC). In addition, we investigated whether SDV affects the depression-like phenotype, spleen weight, synaptic protein expression in the mPFC, and gut microbiota composition in LPS-treated mice.

## Materials and method

### Animals

Forty adult male C57BL/6 mice (aged 8 weeks, body weighing 20–25 g) were purchased from Japan SLC Inc. (Hamamatsu, Japan). Mice were housed under controlled conditions for temperature and humidity with a 12 h light/dark cycle (lights on from 07 to 19 h) and were allowed free access to food (CE-2; CLEA Japan, Inc., Tokyo, Japan) and water. All experiments using mice were carried out in strict accordance with the recommendations in the Guide for the Care and Use of Laboratory Animals of the National Institutes of Health, USA, and were approved by the Chiba University Institutional Animal Care and Use Committee (Permission number: 1-427). Animals were deeply anaesthetized with inhaled isoflurane and rapidly killed by cervical dislocation. All efforts were made to minimize animal suffering.

### Subdiaphragmatic vagotomy (SDV)

Bilateral SDV was performed under anesthesia with 5% isoflurane. Briefly, a 1 cm right transverse abdominal incision was made 0.5 cm below the xiphisternum, starting from the linea alba. The liver was carefully retracted with a small cotton pellet dampened with sterile normal saline and the costal arc was pulled using a vascular clamp, to expose the esophagus. The dorsal and ventral branches of the vagus nerve were exposed along the subdiaphragmatic esophagus under a surgical microscope (Leica, Heidelberg, Germany). Fourteen days after the operation, the observation of an increased stomach size indicated a successful SDV. For sham surgery, the trunk of the vagus nerve was gently exposed but not cut. In all mice that were subjected to SDV, particular care was taken to avoid any injuries to the subdiaphragmatic esophagus. The mice that underwent bilateral SDV were allowed to recover for 14 days.

### Grouping and behavioral tests

**Fig. 1:**
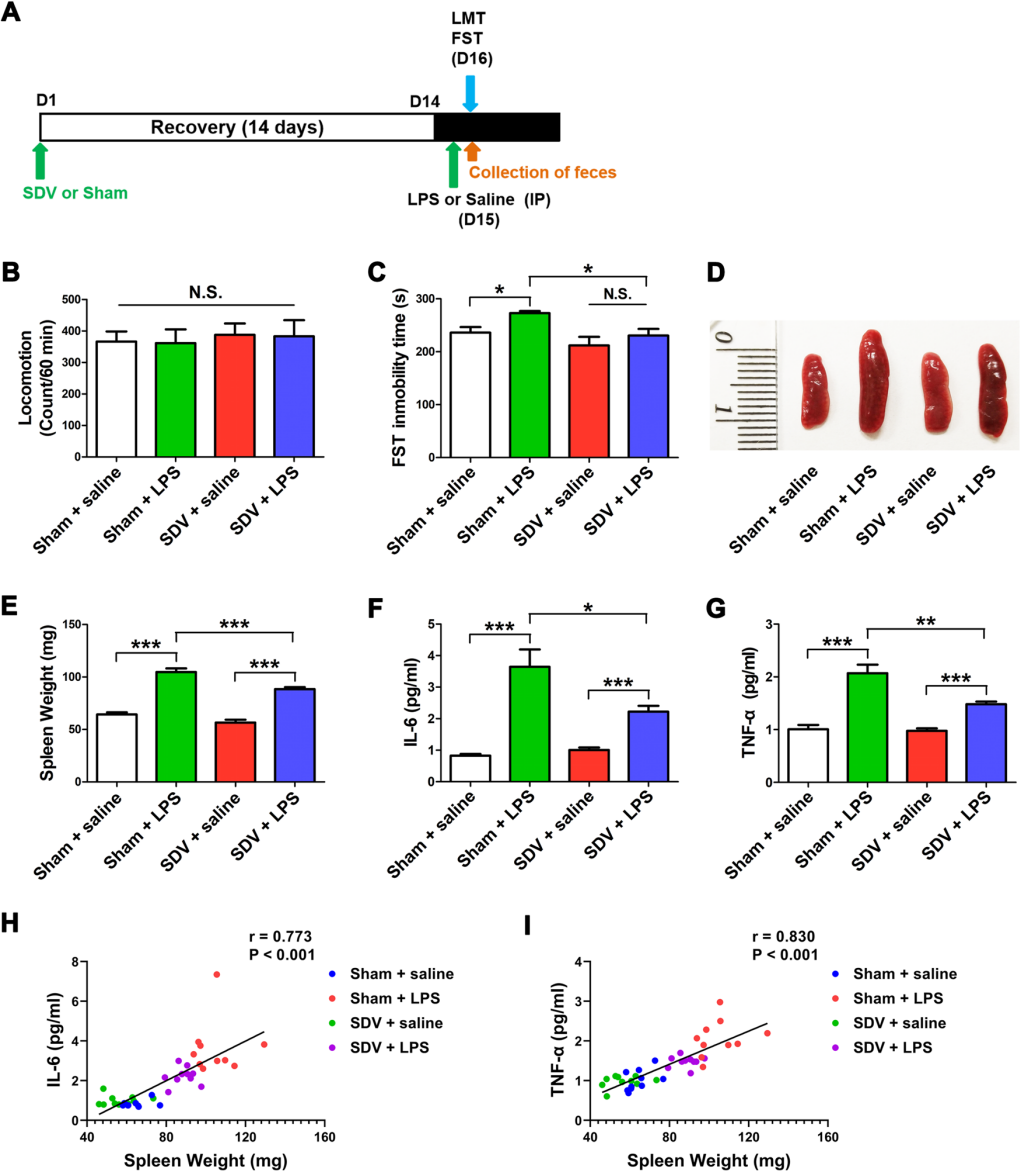
Effects of SDV on depression-like phenotype, spleen weight and inflammatory cytokines after LPS treatment. **a** Treatment schedule. Adult mice were subjected to subdiaphragmatic vagotomy (SDV) and then allowed to recovery for 14 days. On day 15, mice were intraperitoneally (i.p.) injected with lipopolysaccharides (LPS, 0.5 mg/kg) or saline (10 ml/kg). On day 16, fresh feces were collected. Locomotion test and forced swimming test (FST) were performed 22 and 24 h after a single injection of saline or LPS. Blood and brain samples (prefrontal cortex) were collected after behavioral tests (**b**) Locomotion test (LMT). (tow-way ANOVA: LPS: *F*_1,36_ = 0.014, *P* = 0.907; SDV: *F*_1,36_ = 0.271, *P* = 0.606; interaction: *F*_1,36_ = 0.000, *P* = 0.996). **c** FST (two-way ANOVA: LPS: F_1,36_ = 5.666, *P* = 0.0243; SDV: F_1,36_ = 8.126, *P* = 0.0081; interaction: *F*_1,36_ = 0.617, *P* = 0.439). **d** Representative picture of spleen. **e** Spleen weight (two-way ANOVA: LPS: *F*_1,36_ = 197.7, *P* < 0.0001; SDV: *F*_1,36_ = 21.84, *P* < 0.0001; interaction: *F*_1,36_ = 2.756, *P* = 0.106). **f** Plasma levels of IL-6. (two-way ANOVA: LPS: *F*_1,36_ = 58.34, *P* < 0.0001; SDV: *F*_1,36_ = 5.507, *P* = 0.0253; interaction: F_1,36_ = 9.131, *P* = 0.0049). **g** Plasma levels of TNF-α (two-way ANOVA: LPS: *F*_1,36_ = 74.43, *P* < 0.0001; SDV: *F*_1,36_ = 9.243, *P* = 0.0047; interaction: *F*_1,36_ = 9.850, *P* = 0.0036). The data represent mean ± S.E.M. (*n* = 10). *P < 0.05, **P < 0.01, ***P < 0.0001; N.S. not significant. **h** There was a positive correlation (*r* = 0.773, *P* < 0.001) between spleen weight and plasma IL-6. i There was a positive correlation (*r* = 0.830, *P* < 0.001) between spleen weight and plasma TNF-α.

The mice were randomly divided into four groups (*n* = 10/group). Saline (10 ml/kg), or LPS (0.5 mg/kg, Sigma-Aldrich Japan, Tokyo, Japan) was given intraperitoneally (i.p.) to mice subjected to SDV or sham surgery (day 15) (Fig. 1a). The locomotion test (LMT) and forced swimming test (FST) were performed 22 and 24 h after a single injection of saline or LPS.

The mice were deeply anesthetized with inhaled isoflurane (5%) 24 h after the injection of saline or LPS. Blood was collected via cardiac puncture, placed into tubes containing ethylenediaminetetraacetic acid, and immediately centrifuged at 3000 × *g* for 3 min at 4 °C, to obtain plasma, and then stored at −80 °C until bioanalysis. The bilateral mPFC was collected rapidly and stored at −80 °C until bioanalysis. The weight of spleens was recorded immediately after spleen removal^26^.

The LMT and FST were performed as described previously^21,25,48,49^. An automated animal movement analysis system (SCANET MV-40; MELQUEST Co., Ltd, Toyama, Japan) was used to measure the locomotor activity of mice. The cumulative ambulatory activity counts were recorded continuously over a period of 60 min after the mice were placed in the experimental cages (56 cm (length) × 56 cm (width) × 33 cm (height)). The cages were cleaned between the testing sessions. The FST was performed using an automated forced-swim apparatus (SCANET MV-40; MELQUEST Co., Ltd, Toyama, Japan). The mice were individually placed into a cylinder (23 cm (diameter) × 31 cm (height)) with a water depth of 15 cm (water temperature, 23 ± 1 °C). The immobility time was recorded and calculated by the analytical software of the apparatus throughout a 6 min observation time.

### Enzyme-linked immunosorbent assay (ELISA)

The plasma expression levels of IL-6 (Cat Number: 88-7064, Invitrogen, Camarillo, CA, USA) and TNF-α (Cat Number: 88-7324, Invitrogen, Camarillo, CA, USA) were measured using commercial ELISA kits, as per the manufacturer’s instructions.

### Western blotting

Tissue samples from the mPFC and hippocampus were homogenized in ice-cold Laemmli lysis buffer and centrifuged at 3000 × *g* for 10 min at 4 °C, to collect the supernatants. Proteins were quantified using a bicinchoninic acid protein assay kit (Bio-Rad, Hercules, CA). The samples were then mixed with an equal volume of loading buffer (125 mM Tris/HCl, pH 6.8, 20% glycerol, 0.1% bromophenol blue, 10% β-mercaptoethanol, and 4% sodium dodecyl sulfate) and boiled for 5 min at 95 °C. Proteins were separated using 10% sodium dodecyl sulfate polyacrylamide gel electrophoresis (SDS–PAGE) (Mini-PROTEAN^®^ TGX™ Precast Gel; Bio-Rad) and then transferred onto polyvinylidene difluoride membranes using a Trans Blot Mini Cell apparatus (Bio-Rad). The membranes were blocked with 5% skim milk in TBS containing 0.1% Tween 20 (TBST) for 1 h at room temperature, followed by incubation with primary antibodies against PSD-95 (1:1000, Cat Number: 51-6900, Invitrogen, Camarillo, CA, USA), GluA1 (1:1,000, Cat Number: ab31232, Abcam, Cambridge, MA, USA), and β-actin (1:10,000, Sigma-Aldrich Co., Ltd, St Louis, MO, USA) overnight at 4 °C. After three washes with TBST, the membranes were incubated with a horseradish peroxidase-conjugated anti-rabbit or anti-mouse antibody (1:5000) for 1 h at room temperature. After three washes in TBST, the bands were visualized using enhanced chemiluminescence plus the Western Blotting Detection system (GE Healthcare Bioscience) and captured by a ChemiDoc™ Touch Imaging System (170-01401; Bio-Rad Laboratories, Hercules, CA). The images were subjected to grey-scale analysis using the Image Lab^TM^ 3.0 software (Bio-Rad Laboratories).

### Collection of fecal samples and 16S rRNA analysis

Fresh fecal samples of mice were collected before the LMT. The fecal samples were placed into sterilized screw-cap microtubes immediately after defecation and were stored at −80 °C until use. The 16S rRNA analyses of fecal samples were performed at MyMetagenome Co., Ltd (Tokyo, Japan), as reported previously^38,49^.

### Statistical analysis

Data are expressed as the mean ± standard error of the mean (S.E.M). Data were analyzed using two-way analysis of variance (ANOVA) followed by post-hoc Tukey’s multiple comparison tests. Correlation was analyzed by Pearson’s correlation. Significance was set at *P* < 0.05. Statistical analyses were performed using the SPSS version 20.0 software (SPSS, Tokyo, Japan).

## Results

### Effects of SDV on the depression-like phenotype, spleen weight, and inflammatory cytokines after LPS treatment

First, we studied the effects of SDV on the depression-like phenotype, spleen weight, and increased inflammatory cytokines observed in mice after LPS treatment (Fig. 1a). No difference was found in locomotor activity among the four groups (Fig. 1b). A significant difference in the immobility time in the FST, as analyzed by two-way ANOVA, was observed among the four groups (Fig. 1c). LPS significantly increased the immobility time in the FST in sham-operated mice, but not in mice subjected to SDV (Fig. 1c). Interestingly, SDV significantly attenuated the increased immobility time in the FST observed in LPS-treated mice.

A two-way ANOVA of the spleen weight data revealed a significant difference in this parameter among the four groups (Fig. 1d, e). LPS significantly increased the spleen weight in both sham-operated and SVD-operated mice. However, the spleen weight of LPS-treated mice subjected to SDV was significantly lower than that of LPS-treated mice subjected to sham operation.

A two-way ANOVA revealed significant differences in the plasma expression of IL-6 and TNF-α among the four groups (Fig. 1f, g). LPS significantly increased the plasma levels of IL-6 and TNF-α in both sham-operated and SDV-operated mice. However, the plasma levels of IL-6 and TNF-α in the SDV + LPS group were significantly lower than those of the sham + LPS group. Interestingly, positive correlations between spleen weight and plasma IL-6 (or plasma TNF-α) levels were observed in the four groups (Fig. 1h, i). Thus, LPS-induced inflammation in the periphery is associated with increased splenic volume and weight.

### Effects of SDV on the expression of PSD-95 and GluA1 in the brain after LPS treatment

**Fig. 2:**
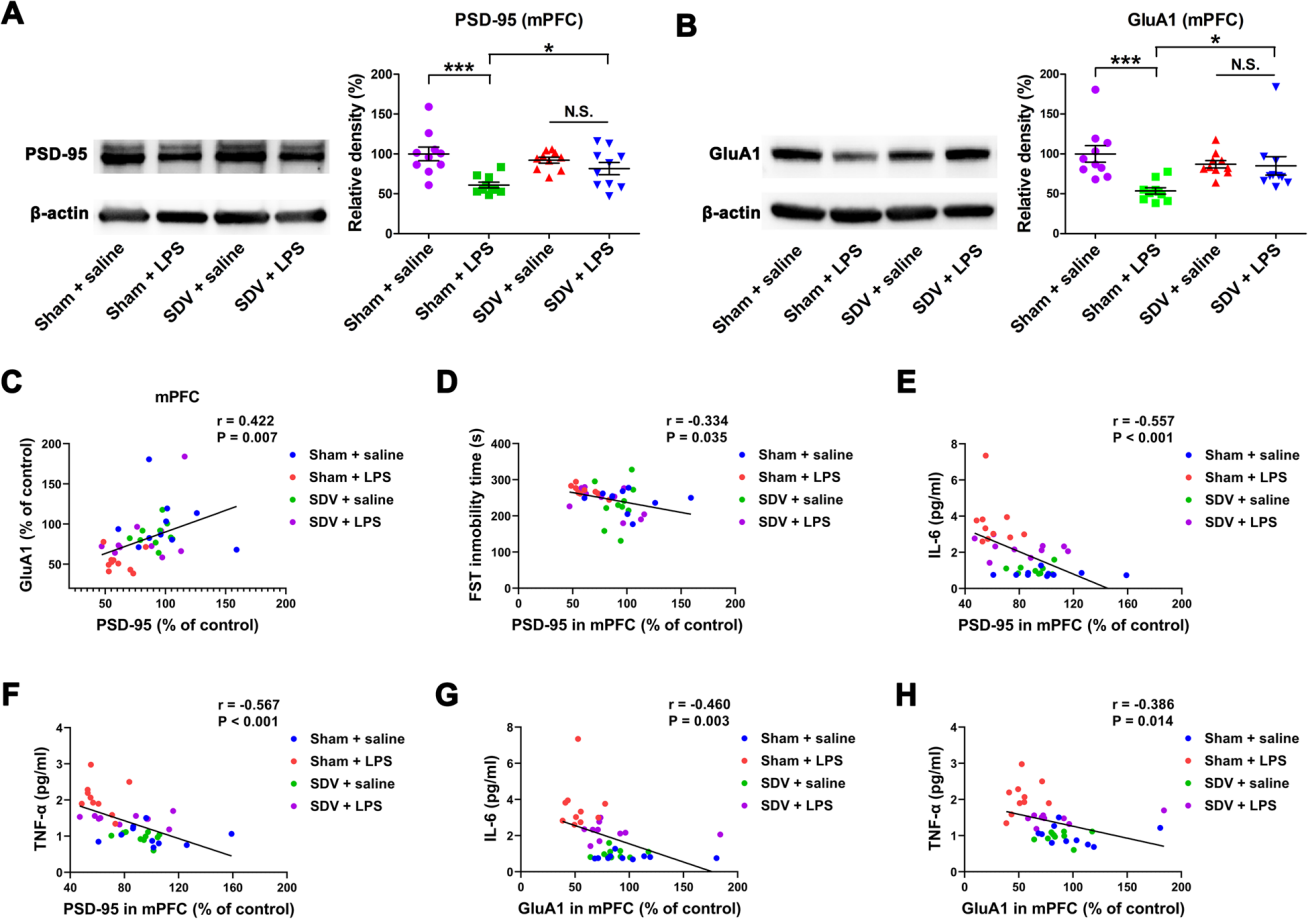
Effects of SDV on protein expression of PSD-95 and GluA1 in the PFC after LPS treatment. **a** Western blot analysis of postsynaptic density-95 (PSD-95) and β-actin in the prefrontal cortex (PFC) of the four groups (two-way ANOVA: LPS: *F*_1,36_ = 15.53, *P* = 0.0004; SDV: *F*_1,36_ = 1.028, *P* = 0.3174; interaction: *F*_1,36_ = 9.131, *P* = 0.0049). **b** Western blot analysis of AMPA receptor subunit GluR1 (GluA1) and β-actin in the PFC of the four groups (LPS: *F*_1,36_ = 8.377, *P* = 0.0064; SDV: *F*_1,36_ = 1.229, *P* = 0.2749; interaction: *F*_1,36_ = 7.055, *P* = 0.0117). The data represent mean ± S.E.M (*n* = 10). *P < 0.05, ***P < 0.0001; N.S. not significant. **c** There was a positive correlation (*r* = 0.422, *P* = 0.007) between PSD-95 and GluA1. **d** There was a negative correlation (*r* = −0.334, *P* = 0.035) between FST immobility time and PSD-95 in the PFC. **e** There was a negative correlation (*r* = −0.557, *P* < 0.001) between PSD-95 in the PFC with plasma IL-6. **f** There was a negative correlation (*r* = −0.567, *P* < 0.001) between PSD-95 in the PFC and plasma TNF-α. **g** There was a negative correlation (*r* = −0.460, *P* = 0.003) between GluA1 in the PFC and plasma IL-6. **h** There was a negative correlation (*r* = −0.386, *P* = 0.014) between GluA1 in the PFC and plasma TNF-α.

Two-way ANOVA showed a significant difference in the levels of expression of the PSD-95 and GluA1 proteins in the mPFC among the four groups (Fig. 2a, b). LPS significantly decreased the expression of PSD-95 and GluA1 in the mPFC in sham-operated mice, but not in mice subjected to SDV.

Positive correlations were detected between PSD-95 and GluA1 in the mPFC of the four groups (Fig. 2c). Furthermore, there was a negative correlation between the immobility time in the FST and PSD-95 levels in the mPFC in the four groups (Fig. 2d). In turn, there were negative correlations between plasma IL-6 (or TNF-α) levels and PSD-95 (or GluA1) expression in the mPFC in the four groups (Fig. 2e–h). Thus, LPS-induced inflammation is associated with the depression-like phenotype and reduced expression of synaptic proteins in the mPFC.

### Composition of gut microbiota

**Fig. 3:**
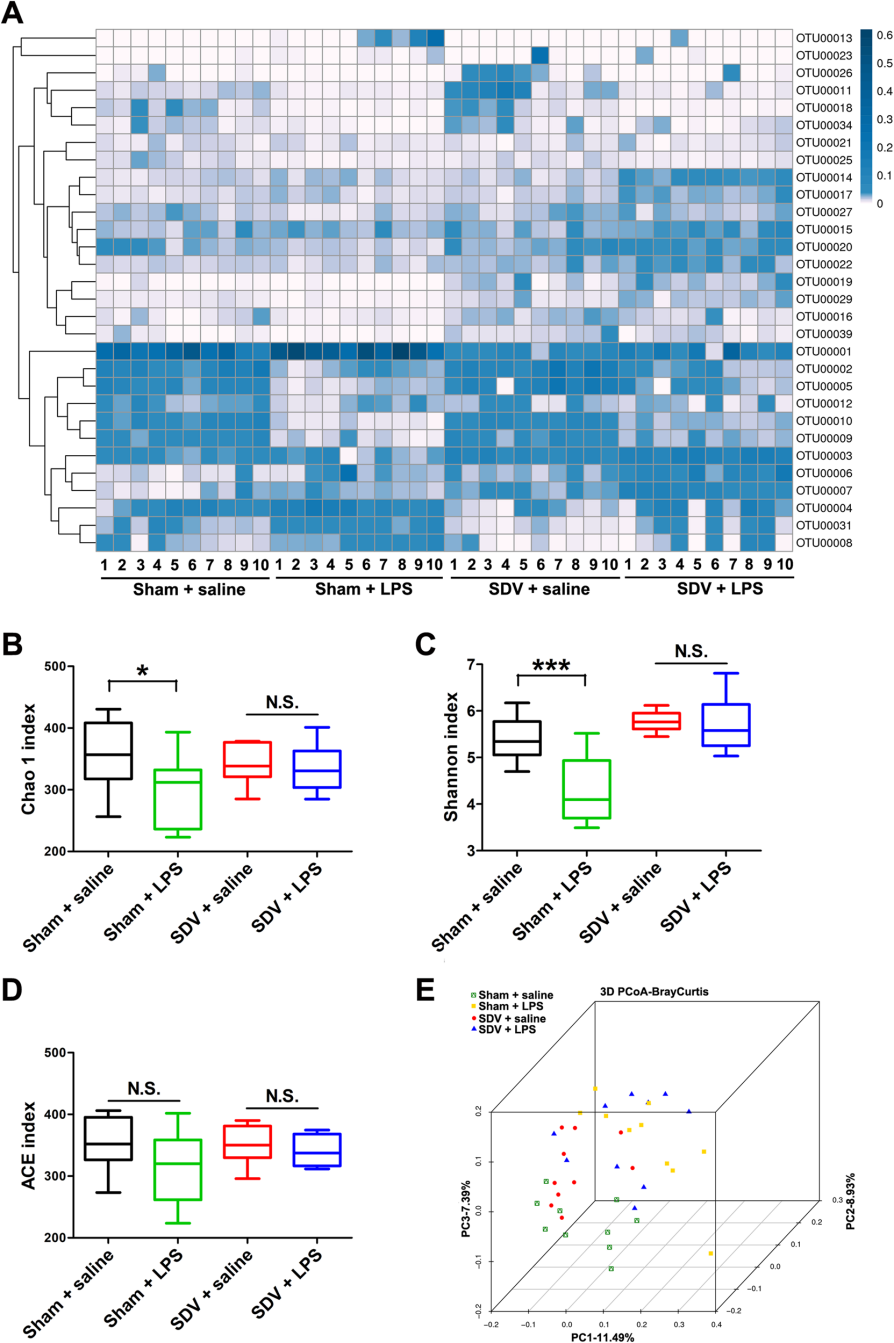
α-diversity and β-diversity in gut microbiota. **a** Heat map of differential levels of bacteria between the four groups. **b** Chao1 index. **c** Shannon index. **d** ACE index. **e** PCoA analysis of gut bacteria data (Bray–Curtis dissimilarity). The data represent mean ± S.E.M. (*n* = 10). *P < 0.05, ***P < 0.0001; N.S. not significant.

The composition of the gut microbiota was investigated in the four groups. α-diversity, defined as the gut microbiota richness, can be measured using different indices, including the Chao1 index, Shannon index, and ACE index. A two-way ANOVA revealed significant differences in the Chao1 and Shannon indices among the four groups (Fig. 3a–c). A two-way ANOVA showed an absence of significant differences in the ACE index among the four groups (Fig. 3d). Regarding β-diversity, principal coordinate analysis plots of Bray–Curtis dissimilarity between the four groups showed that the dots representing the sham + LPS group were far from the dots representing the other three groups (Fig. 3e).

**Fig. 4:**
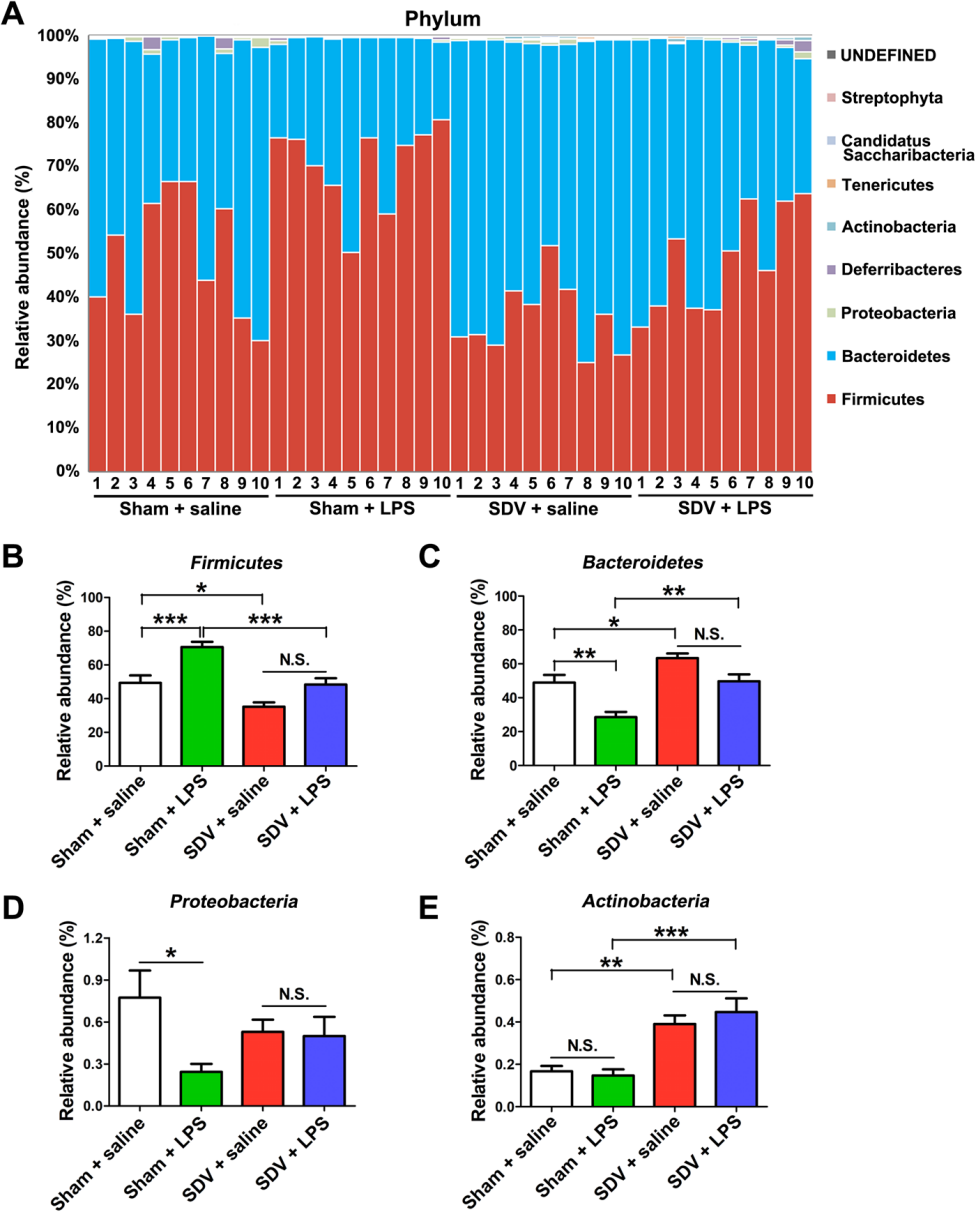
Altered gut bacteria composition at the phylum level. **a** Relative abundance at the phylum level in the four groups. **b***Firmicutes* (two-way ANOVA: LPS: *F*_1,36_ = 24.11, *P* < 0.0001; SDV: *F*_1,36_ = 26.94, *P* < 0.0001; interaction: *F*_1,36_ = 1.324, *P* = 0.2575). **c***Bacteroidetes* (two-way ANOVA: LPS: *F*_1,36_ = 21.37, *P* < 0.0001; SDV: *F*_1,36_ = 23.49, *P* < 0.0001; interaction: *F*_1,36_ = 0.853, *P* = 0.3619). **d***Proteobacteria* (two-way ANOVA: LPS: *F*_1,36_ = 4.683, *P* = 0.0372; SDV: *F*_1,36_ = 0.003, *P* = 0.9592; interaction: *F*_1,36_ = 3.733, *P* = 0.0612). **e***Actinobacteria* (two-way ANOVA: LPS: *F*_1,36_ = 0.180, *P* = 0.6739; SDV: *F*_1,36_ = 36.67, *P* < 0.0001; interaction: *F*_1,36_ = 0.787, *P* = 0.3809). The data represent mean ± S.E.M. (*n* = 10). *P < 0.05, **P < 0.01, ***P < 0.0001; N.S. not significant.

At the phylum level, *Firmicutes* were most abundant in the sham + LPS group (Fig. 4a, b). The abundance of *Firmicutes* was more pronounced in the sham + LPS group than it was in the sham + saline and SDV + LPS groups (Fig. 4b). In contrast, *Bacteroidetes* were also an abundant phylum in these samples. The abundance of *Bacteroidetes* was lower in the sham + LPS group than it was in the sham + saline and SDV + LPS groups (Fig. 4c). The levels of *Proteobacteria* in the sham-operated mice were decreased after treatment with LPS (Fig. 4d), whereas *Actinobacteria* levels in the sham and SDV-operated mice were not altered after treatment with LPS (Fig. 4e). Moreover, there were no differences in the abundance of *Firmicutes*, *Bacteroidetes*, *Proteobacteria*, and *Actinobacteria* between the SDV + saline group and the SDV + LPS group (Fig. 4b–e). Interestingly, the abundance of *Firmicutes* in the SDV + saline group was lower than that in the sham + saline group, whereas the abundance of *Bacteroidetes* and *Actinobacteria* was higher in the SDV + saline group than it was in the sham + saline group (Fig. 4b–d).

**Fig. 5:**
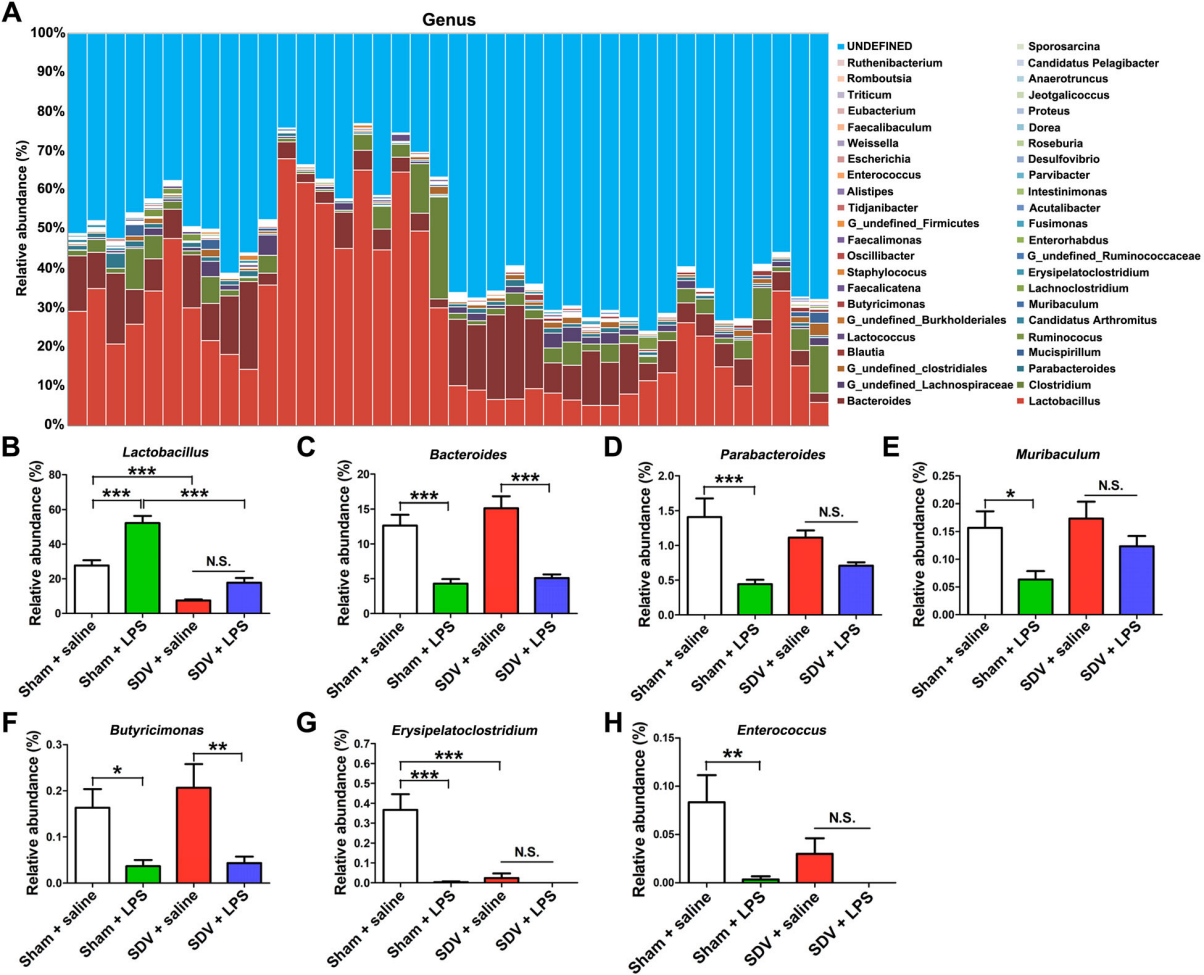
Altered gut bacteria composition at the genus level. **a** Relative abundance at the genus level in the four groups. **b***Lactobacillus* (two-way ANOVA: LPS: *F*_1,36_ = 34.85, *P* < 0.0001; SDV: F_1,36_ = 85.80, *P* < 0.0001; interaction: *F*_1,36_ = 5.862, *P* = 0.0206). **c***Bacteroides* (two-way ANOVA: LPS: *F*_1,36_ = 57.36, *P* < 0.0001; SDV: *F*_1,36_ = 1.833, *P* = 0.1842; interaction: *F*_1,36_ = 0.483, *P* = 0.4917). **d***Parabacteroides* (two-way ANOVA: LPS: *F*_1,36_ = 21.64, *P* < 0.0001; SDV: *F*_1,36_ = 0.010, *P* = 0.9194; interaction: *F*_1,36_ = 3.659, *P* = 0.0637). **e***Muribaculum* (two-way ANOVA: LPS: *F*_1,36_ = 8.636, *P* = 0.0057; SDV: *F*_1,36_ = 2.471, *P* = 0.1247; interaction: *F*_1,36_ = 0.789, *P* = 0.3802). **f***Butyricimonas* (two-way ANOVA: LPS: *F*_1,36_ = 18.24, *P* = 0.0001; SDV: *F*_1,36_ = 0.542, *P* = 0.4663; interaction: *F*_1,36_ = 0.292, *P* = 0.5925). **g***Erysipelatoclostridium* (two-way ANOVA: LPS: *F*_1,36_ = 22.22, *P* < 0.0001; SDV: *F*_1,36_ = 17.86, *P* = 0.0002; interaction: *F*_1,36_ = 17.18, *P* = 0.0002). **h***Enterococcus* (two-way ANOVA: LPS: *F*_1,36_ = 11.36, *P* = 0.0018; SDV: *F*_1,36_ = 3.014, *P* < 0.0911; interaction: *F*_1,36_ = 2.346, *P* = 0.1343). The data represent mean ± S.E.M. (*n* = 10). *P < 0.05, **P < 0.01, ***P < 0.0001; N.S. not significant.

At the genus level, both LPS treatment and SDV altered the fecal microbiota composition (Fig. 5a). LPS significantly increased *Lactobacillus* abundance in the sham-operated mice, but not in the SDV-operated mice (Fig. 5b). In contrast, LPS significantly decreased the abundance of *Bacteroides*, *Parabacteroides*, *Muribaculum*, *Butyricimonas*, *Erysipelatoclostridium*, and *Enterococcus* in the sham-operated mice (Fig. 5c–h). However, LPS did not alter the abundance of *Parabacteroides*, *Muribaculum*, *Erysipelatoclostridium*, and *Enterococcus* in the SDV-operated mice (Fig. 5d, e, g, h).

**Fig. 6:**
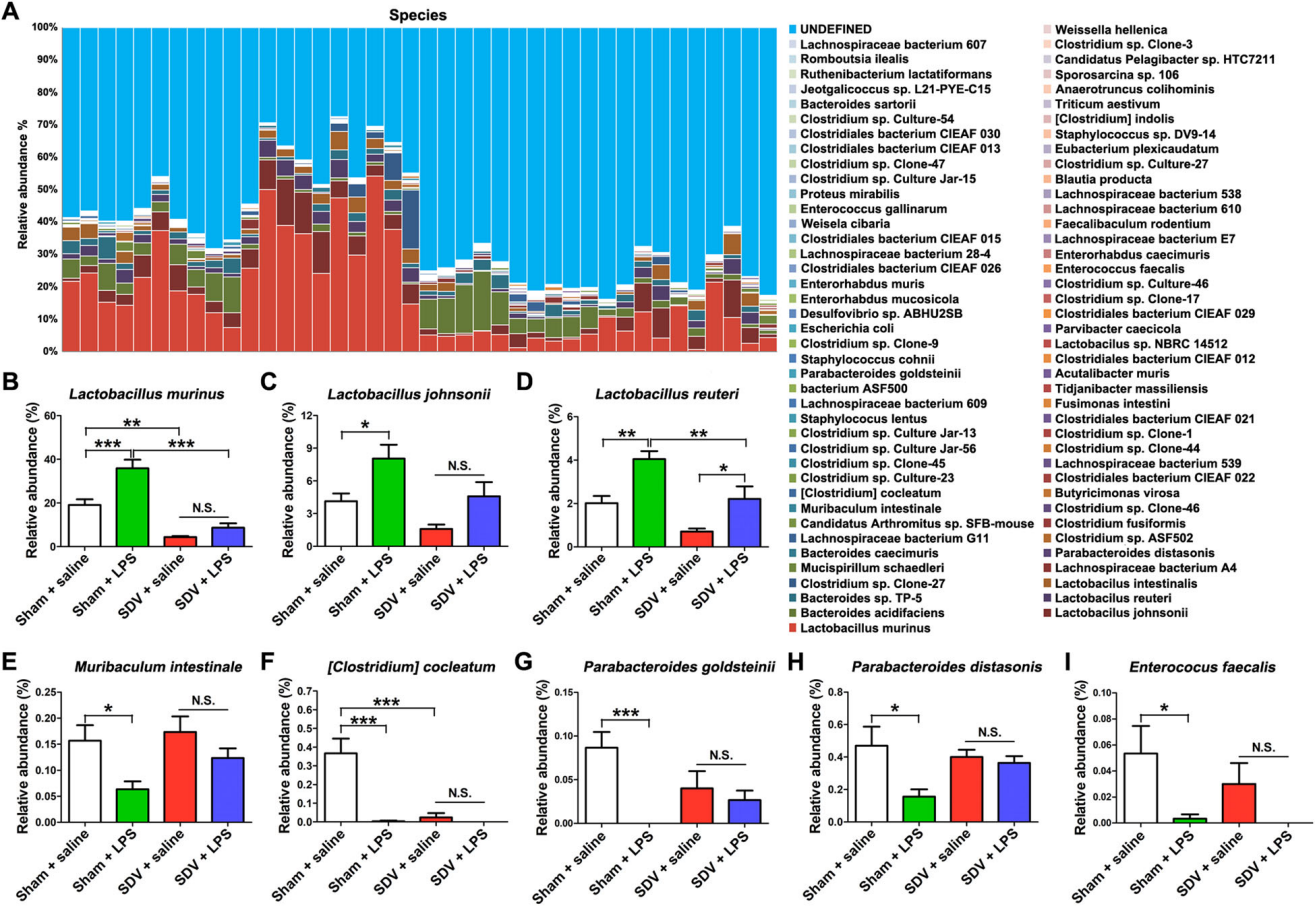
Altered gut bacteria composition at the species level. **a** Relative abundance at the species level in the four groups. **b***Lactobacillus murinus* (two-way ANOVA: LPS: *F*_1,36_ = 16.64, *P* = 0.0002; SDV: *F*_1,36_ = 66.09, *P* < 0.0001; interaction: *F*_1,36_ = 5.849, *P* = 0.02085). **c***Lactobacillus johnsonii* (two-way ANOVA: LPS: *F*_1,36_ = 12.13, *P* = 0.0013; SDV: *F*_1,36_ = 9.196, *P* = 0.0045; interaction: *F*_1,36_ = 0.212, *P* = 0.6479). **d***Lactobacillus reuteri*. (two-way ANOVA: LPS: *F*_1,36_ = 21.04, *P* < 0.0001; SDV: *F*_1,36_ = 16.75, *P* = 0.0012; interaction: *F*_1,36_ = 0.469, *P* = 0.4977). **e***Muribaculum intestinale* (two-way ANOVA: LPS: *F*_1,36_ = 8.636, *P* = 0.0057; SDV: *F*_1,36_ = 2.471, *P* = 0.1247; interaction: *F*_1,36_ = 0.789, *P* = 0.3802). **f***[Clostridium] cocleatum* (two-way ANOVA: LPS: *F*_1,36_ = 22.22, *P* < 0.0001; SDV: *F*_1,36_ = 17.86, *P* = 0.0002; interaction: *F*_1,36_ = 17.18, *P* = 0.0002). **g***Parabacteroides goldsteinii* (two-way ANOVA: LPS: *F*_1,36_ = 11.98, *P* = 0.0014; SDV: *F*_1,36_ = 0.479, *P* = 0.4932; interaction: *F*_1,36_ = 6.444, *P* = 0.0156). **h***Parabacteroides distasonis* (two-way ANOVA: LPS: *F*_1,36_ = 6.288, *P* = 0.0168; SDV: *F*_1,36_ = 0.959, *P* = 0.3340; interaction: *F*_1,36_ = 3.929, *P* = 0.0551). **i***Enterococcus faecalis* (two-way ANOVA: LPS: *F*_1,36_ = 8.907, *P* = 0.0051; SDV: *F*_1,36_ = 0.990, *P* = 0.3265; interaction: *F*_1,36_ = 0.557, *P* = 0.4604). The data represent mean ± S.E.M. (*n* = 10). *P < 0.05, **P < 0.01, ***P < 0.0001; N.S. not significant.

The composition of gut microbiota at the species level in the four groups is shown in Fig. 6a. The abundance of *Lactobacillus murinus*, *Lactobacillus johnsonii*, and *Lactobacillus reuteri* in the sham-operated mice was increased after LPS administration, whereas the abundance of *Muribaculum intestinale*, *[Clostridium] cocleatum*, *Parabacteroides goldsteinii*, *Parabacteroides distasonis*, and *Enterococcus faecalis* in the sham-operated mice was decreased after LPS treatment (Fig. 6). Interestingly, there were no differences in the abundance of *L. murinus*, *L. johnsonii*, *M. intestinale*, *[C.] cocleatum*, *P. goldsteinii*, *P. distasonis*, and *E. faecalis* between the SDV + saline group and the SDV + LPS group (Fig. 6).

### Correlations between spleen weight and the abundance of gut microbiota

**Fig. 7:**
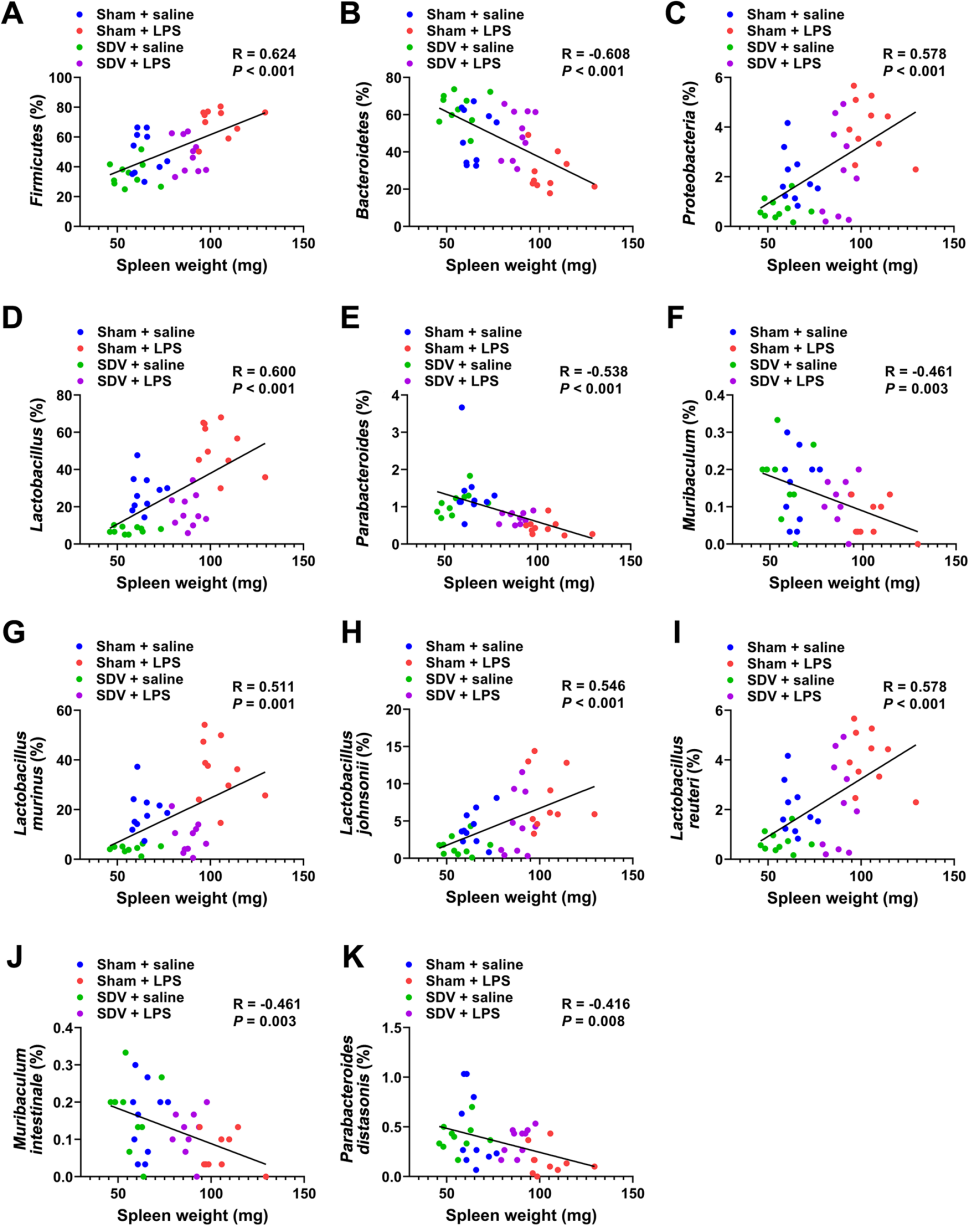
Pearson correlation analysis of spleen weight and gut microbiota. There was a positive correlation between spleen weight and *Firmicutes* (*r* = 0.624, *P* < 0.001) (**a**), *Proteobacteria* (*r* = 0.578, *P* < 0.001) (**c**), *Lactobacillus* (*r* = 0.600, *P* < 0.001) (**d**), *Lactobacillus murinus* (*r* = 0.511, *P* = 0.001) (**g**), *Lactobacillus johnsonii* (*r* = 0.546, *P* < 0.001) (**h**), *Lactobacillus reuteri* (*r* = 0.578, *P* < 0.001) (**i**). There was a negative correlation between spleen weight and *Bacteroidetes* (*r* = −0.608, *P* < 0.001) (**b**), *Parabacteroides* (*r* = −0.538, *P* < 0.001) (**e**), *Muribaculum* (*r* = −0.461, *P* = 0.003) (**f**), *Muribaculum intestinale* (*r* = −0.461, *P* = 0.003) (**j**), *Parabacteroides distasonis* (*r* = −0.416, *P* = 0.008) (**k**).

There were positive correlations between spleen weight and the abundance of *Firmicutes*, *Proteobacteria*, *Lactobacillus*, *L. murinus*, *L. johnsonii*, and *L. reuteri* among the four groups (Fig. 7a, c, d, g, h, i). In contrast, there were negative correlations between spleen weight and the abundance of *Bacteroidetes*, *Parabacteroides*, *Muribaculum*, *M. intestinale*, and *P. distasonis* among the groups (Fig. 7b, e, f, j, k).

## Discussion

The major findings of this study were as follows. First, LPS (0.5 mg/kg) caused a depression-like phenotype, inflammation, and downregulation of synaptic proteins (i.e., PSD-95 and GluA1) in the mPFC in the sham-operated mice, which was consistent with previous studies^21,24,25^. In contrast, LPS did not produce a depression-like phenotype or downregulation of synaptic proteins in the mPFC of SDV-operated mice. Furthermore, we found a notable increase in spleen weight in the sham-operated mice after LPS administration. Interestingly, SDV significantly attenuated the increased spleen weight and plasma expression of IL-6 and TNF-α detected in mice after LPS administration. Collectively, these results suggest that the subdiaphragmatic vagus nerve plays a role in the depression-like phenotype, inflammation, increases in spleen volume, and reduced synaptic proteins in the PFC observed after a single administration of LPS. Interestingly, we found positive correlations between the plasma levels of IL-6 (or TNF-α) and spleen weight, suggesting that LPS-induced inflammatory events play a role in the increase in spleen weight. The results of the 16S rRNA analysis suggest that LPS caused significant changes in the diversity of the host gut microbiota in sham-operated mice, but not in SDV-operated mice. In an unweighted UniFrac PCoA, the dots representing the sham + LPS group were located far away from the dots representing the other three groups. Moreover, we found correlations between spleen weight and the abundance of the components of the microbiome, suggesting a relationship within the spleen–inflammation–microbiome axis. Taken together, our results indicate that LPS might produce a depression-like phenotype, increase spleen weight, trigger systemic inflammation, downregulate synaptic proteins in the mPFC, and cause abnormal composition of gut microbiota in mice through the brain–gut–microbiota axis and brain–spleen axis via the subdiaphragmatic vagus nerve.

Previous studies demonstrated that the ingestion of beneficial bacteria or selective serotonin reuptake inhibitors alleviated stress-induced anxiety and depression-like behaviors via the subdiaphragmatic vagus nerve, and that these antidepressant-like effects were abolished after SDV^46,50^. It was also reported that SDV blocked the depression-like phenotype after intraperitoneal injection of LPS (0.25 mg/kg) in rats^47^, which is consistent with the present results. Here, we found that LPS produced a depression-like phenotype, an increase in plasma IL-6 and TNF-α levels, and downregulation of synaptic proteins in the mPFC in the sham-operated mice, in agreement with previous reports^20,21,23,25^. In contrast, LPS did not cause a depression-like phenotype or reduced synaptic proteins in the mPFC of the SDV-operated mice. Thus, the subdiaphragmatic vagus nerve seems to play a role in the depression-like phenotype and alteration of synaptic protein expression detected in LPS-treated mice. Although the detailed mechanisms underlying the depression-like phenotype of the sham-operated mice caused by LPS are currently unknown, this study suggests that the subdiaphragmatic vagus nerve is necessary for the LPS-induced depression-like phenotype and the downregulation of synaptic proteins in the mPFC. In contrast, it is well known that LPS-induced inflammation is a dynamic process, from induction to recovery^2^. A further detailed study of the effects of the subdiaphragmatic vagus nerve on the different stages of LPS-induced systemic inflammation and neuroinflammation is needed.

Recently, we reported that CSDS significantly increased the expression of IL-6 in the blood of water-treated mice, but not in that of antibiotic-treated mice, suggesting that antibiotic-induced microbiota depletion has anti-inflammatory effects^38^. Furthermore, CSDS significantly reduced the expression of the synaptic proteins in the PFC of the water-treated mice, but not of the antibiotic-treated mice. These data suggest that antibiotic-induced depletion of gut bacteria leads to stress resilience in CSDS-exposed mice via the brain–gut–microbiota axis^38^. In this study, LPS caused a depression-like phenotype and an abnormality in gut microbiota composition through systemic inflammation in the sham-operated mice, whereas LPS did not cause these changes in SDV-operated mice. These data suggest that the brain–gut–microbiota axis via the subdiaphragmatic vagus nerve exerts effects on the development of the depression-like phenotype in LPS-treated mice, although the precise underlying mechanisms remain unclear. Further detailed studies are required to clarify the relationship between the brain–gut–microbiota axis via the subdiaphragmatic vagus nerve and inflammation-related behavioral abnormalities.

Recently, we observed marked increases in spleen weight in CSDS-susceptible mice compared with non-CSDS control mice and CSDS-resilient mice^26^. In addition, McKim et al.^51^ reported that the total increase in the number of splenic erythrocytes, monocytes, and granulocytes derived from splenic progenitors that migrated from the bone marrow is associated with an increase of about twofold in the size and weight of the spleen after chronic stress. In this study, we also found that LPS caused an increase in spleen weight in the sham-operated mice, and that SDV significantly alleviated the LPS-induced increase in spleen weight. In addition, it has been reported that repeated LPS (0.1 mg/kg/day for 10 days) administration increased spleen weight in rats^52^. Interestingly, we found positive correlations between IL-6 (or TNF-α) and spleen weight, indicating a close relationship between peripheral inflammation and spleen weight. It is also known that LPS produces excessive lymphocyte proliferation and induces large-scale pro-inflammatory cytokine production, resulting in spleen enlargement. Given the role of the immune system in the pathogenesis of depression, these data suggest that the brain–spleen axis via the subdiaphragmatic vagus nerve may exert effects on the depression-like phenotype, increase in spleen weight, and inflammation in the periphery observed in the LPS-treated mice. Further detailed studies are required to confirm the relationship between the brain–spleen axis via the subdiaphragmatic vagus nerve and inflammation-related behavioral abnormalities.

Several routes, including the vagus nerve, the immune system, and the enteric nervous system, mediate the bidirectional communication between the brain and the gut microbiota^40–44^. In this study, we found that LPS did not produce a depression-like behavior or reduced synaptic protein expression in the mPFC in the vagotomized mice. We speculate that the dominant specific microorganisms present in the intestinal flora after LPS treatment may lead to the depression-like phenotype through the subdiaphragmatic vagus nerve system, although further study is needed to confirm this hypothesis. The subdiaphragmatic vagus nerve may mediate the communication between the brain and the gut microbiota to exert beneficial or detrimental effects depending on the predominance of probiotic or pathogenic microorganisms present in the intestinal flora. Interestingly, transcutaneous auricular vagus nerve stimulation therapy is approved for patients with treatment-resistant depression^53–55^, suggesting the anti-inflammatory properties of vagus nerve stimulation^56^. Taken together, these findings suggest that the vagus nerve plays a key role in depression, in the antidepressant actions of certain compounds, and in vagus nerve stimulation. Furthermore, it seems that the different branches of the vagus nerve play different roles under pathological conditions.

Recently, we reported that levels of colony stimulating factor 1 receptor (CSF1R) in the spleen, but not postmortem brain, from patients with depression were significantly lower than those of control groups, and that there was a negative correlation between CSF1R and interacting protein SPI1 in the spleen^57^, suggesting a brain–spleen axis in psychiatric disorders such as depression^26,57,58^. A recent study demonstrated that neurons in the central nucleus of the amygdala and the paraventricular nucleus that express corticosterone-releasing hormone are connected to the splenic nerve, indicating a key role of brain–spleen communication in antibody production^59^. Collectively, it is likely that brain–spleen axis plays a key role in a number of disorders related with immune system.

In conclusion, the present study showed that LPS produced a depression-like phenotype and caused abnormal composition of gut microbiota in mice via the subdiaphragmatic vagus nerve. It is likely that both the brain–gut–microbiota axis and the brain–spleen axis via the subdiaphragmatic vagus nerve play an important role in the pathogenesis of inflammation-related depression. Future studies of the role of the subdiaphragmatic vagus nerve in the brain–gut–microbiota axis and the brain–spleen axis in other inflammatory-mediator-induced depression models or noninflammatory depression models (i.e., CSDS) are needed.
